# Designing of Chitosan Derivatives Nanoparticles with Antiangiogenic Effect for Cancer Therapy

**DOI:** 10.3390/nano10040698

**Published:** 2020-04-07

**Authors:** Oana-Maria Dragostin, Rodica Tatia, Sangram Keshari Samal, Anca Oancea, Alexandra Simona Zamfir, Ionuț Dragostin, Elena-Lăcrămioara Lisă, Constantin Apetrei, Carmen Lăcrămioara Zamfir

**Affiliations:** 1 Research Centre in the Medical-Pharmaceutical Field, Faculty of Medicine and Pharmacy, “Dunarea de Jos” University of Galati, 800008 Galati, Romania; elenalysa@yahoo.com; 2 Romanian National Institute of Research and Development for Biological Sciences, 296 Splaiul Independentei, 060031 Bucharest, Romania; rodica.tatia@gmail.com (R.T.); oancea.anca@gmail.com (A.O.); 3 Laboratory of Biomaterials and Regenerative Medicine for Advanced Therapies, Indian Council of Medical Research-Regional Medical Research Center, Bhubaneswar-751 023, Odisha, India; sksamalrec@gmail.com; 4 Department of Morpho-Functional Sciences I, Faculty of Medicine, University of Medicine and Pharmacy “Gr.T.Popa”, 700115 Iasi, Romania; simona.zamfir14@gmail.com (A.S.Z.); ionut.dragostin@yahoo.com (I.D.); zamfircia@yahoo.com (C.L.Z.); 5 Department of Chemistry, Physics and Environment, The European Centre of Excellence for the Environment, Faculty of Sciences and Environment, “Dunarea de Jos” University of Galati, 800008 Galati, Romania

**Keywords:** angiogenesis, nanoparticles, chitosan derivatives, chorioallantoic membrane, cancer therapy, antioxidant action

## Abstract

Angiogenesis is a physiological process involving the growth of new blood vessels, which provides oxygen and required nutrients for the development of various pathological conditions. In a tumor microenvironment, this process upregulates the growth and proliferation of tumor cells, thus any stage of angiogenesis can be a potential target for cancer therapies. In the present study, chitosan and his derivatives have been used to design novel polymer-based nanoparticles. The therapeutic potential of these newly designed nanoparticles has been evaluated. The antioxidant and MTT assays were performed to know the antioxidant properties and their biocompatibility. The in vivo antiangiogenic properties of the nanoparticles were evaluated by using a chick Chorioallantoic Membrane (CAM) model. The obtained results demonstrate that chitosan derivatives-based nanostructures strongly enhance the therapeutic effect compared to chitosan alone, which also correlates with antitumor activity, demonstrated by the in vitro MTT assay on human epithelial cervical Hep-2 tumor cells. This study opens up new direction for the use of the chitosan derivatives-based nanoparticles for designing of antiangiogenic nanostructured materials, for future cancer therapy.

## 1. Introduction

Angiogenesis is the development of new blood vessels, a process that normally occurs during embryogenesis, wound healing, or uterus function. Besides these normal situations, there are those cases of pathologic angiogenesis found in: diabetic retinopathy, chronic inflammation, ischemic heart, and cancer. With the increase of cancer prevalence among the global population, the discovery of new anti-cancer therapeutics has not been at the same rate. 

While stimulation of angiogenesis in cancer leads to metastasis, the anti-angiogenic therapy proved to be an important approach against tumor growth. In this respect, it is a well-known fact that the consumption of antioxidants can be recommended to achieve the inhibition of angiogenesis or reversal of unwanted cell–cell adherence [1]. Several studies have been conducted to investigate the connection between antioxidants and pathological angiogenesis, thus leading to mixed conclusions from studies that have been associated with a lower total cancer incidence with the use of antioxidants [2], to studies that have highlighted the lack of any benefit of using antioxidants on the incidence of cancer [3]. All these results are, for certain ones, closely related with the type of antioxidant included in the study (alpha tocopherol, beta-carotene, ascorbic acid) and the investigated tumor location and not least the investigated pharmaceutical formulation. In this context, the use of antioxidants in the form of nanoparticles could improve the efficiency of this therapy due to specific surface area of nanostructures [4], thereby ensuring better contact with cells that would increase the chances of pathological angiogenesis inhibition. However, to have more success in cancer therapy, more studies should be conducted in this direction. 

Chitosan is a natural cationic polymer exhibits three chemical reactive sites including a primary amine and two primary or secondary hydroxyl groups for further modification [5,6,7]. Nano/microparticles coated with chitosan, synthesized through different techniques for encapsulation (chemical, physical and mechanical techniques), are in continuous development, aiming at the entrapment of bioactive substances, enzymes, hormones, antimicrobial agents, vitamins, minerals, antioxidant agents, and nutrients [8,9]. The benefits of this action are: components separation, which assures the prevention of drug incompatibility, substances protection against acids, alkalis, heat, ultraviolet rays, and oxidant agents, leading to extension of validity. For this purpose, vitamin A has been encapsulated to protect it from oxidation; similarly, probiotics have been encapsulated to be protected from digestive juices [8], while paracetamol (acetaminophen) was encapsulated for masking the bitter taste of it [10]. In addition, there are studies in the literature that have highlighted the role of chitosan nanoparticles on pathological angiogenesis [11], but this was done without studying the involvement of their antioxidant action in this process and emphasizing only the role of nanoparticles as drug delivery systems [12,13]. Our studies carried out by now [14,15] have already demonstrated the high antioxidant potential of chitosan and its similar derivatives. In addition to the existing results, the purpose of the present work is to develop and validate new nanoparticles based on chitosan low molecular weight derivatives, taking into account not only their role as carriers but their action itself and their antioxidant potential which is beneficial in inhibiting angiogenesis, as discussed above.

Different methods for in vivo evaluation of angiogenesis and antiangiogenesis have already been analyzed in the specialized literature [16]. The CAM (Chick Chorioallantoic Membrane) model allows the assessment of the degree of angiogenesis, as well as the quality of the newly formed vessels, when intervening vascular endothelial growth factors (VEGF) or conversely, factors that inhibit the development of new blood vessels, such as antiangiogenic drugs. Since the development of tumors is dependent on angiogenesis, the purpose of anticancer therapy is the occlusion of blood vessels that eventually feed the tumor. The efficiency of formulations containing antiangiogenic drugs, such as nanoparticles in our case, using the CAM model, can be easily performed by evaluating the occlusion of blood vessels [17].

Angiogenesis inhibitors are part of anticancer drugs that appear to have low haematological toxicity, and in the future to further reduce the frequency and severity of adverse reactions, delivery of antiangiogenic factors to the tumor site is expected by using genetically modified bacteria (such as *Clostridium, Bifidobacteria* and *Salmonella*) that are capable of colonizing solid tumors in vivo [18]. On the other hand, derivatives with a sulfonamide structure, having anticancer properties, have already been highlighted in the literature. These anti-cancer therapeutics, act by inhibiting Wnt (Wingless-related integration site) transcription pathway [19]. The use of chitosan-sulfonamide derivatives to prepare nanoparticles for increasing the inhibition capacity of angiogenesis could prove to be an important strategy in future antitumor therapy.

## 2. Materials and Methods 

### 2.1. Design of Chitosan-Derived Nanoparticles 

Materials: Chitosan with low molecular weight (CL, 100 kDa, deacetylation degree >85%), acetic acid, sodiumhydroxide, sodium tripolyphosphate (TPP) and organic solvents(p.a.) were purchased from Sigma Aldrich Company. All solvents and chemicals had a high degree of purity.

In continuation to our previous studies carried out on chitosan [14,15], this paper purpose has as a starting point the use of four previously obtained chitosan-sulfonamide derivatives; note here with CLA, CLB, CLC, and CLD (where CLA- *Chitosan-sulfamethoxydiazine*, CLB- *Chitosan-sulfadiazine*, CLC- *Chitosan-sulfadimethoxine*, and CLD- *Chitosan-sulfisoxazole*) to obtain innovative nanoparticles’ formulations. The main goal will be to manufacture the nanoparticles by using ionic reticulation as a cross-linking agent sodium tri-polyphosphate (STPP). In this manner, the preparation method will be optimized using pure chitosan, and the established parameters will be further used to obtain nanoparticles based on chitosan low molecular weight derivatives. A similar method was used in one of the previous studies, in obtaining chitosan micro-particles loaded with xanthine derivatives for improvement of type 2 diabetes [20].

Methods of obtaining nanoparticles involves the following technique: each 1 mL 0.1% chitosan derivative solution was dropwise and stirred at 700 rpm in a 10 mL 0.005% TPP (sodium tripolyphosphate) aqueous solution. The wet phase of nanoparticles were analyzed using a Malvern Autosizer 4800 Malvern Instruments, Worcestershire, UK, DLS spectrometer (Dynamic Light Scattering spectroscopy) [21]. For later use of the nanoparticles, these were separated by centrifugation, washed with distilled water, and dried by lyophilization, using a vertical freeze dryer model BK-FD12S/-55.

### 2.2. Spectral Characterization of Nanoparticles: FT-IR Analysis

The infrared measurements were acquired with a Bruker ALPHA FT-IR spectrophotometer (BrukerOptik GmbH, Ettlingen, Germany) in the spectral region of 4000–500 cm^−1^ [22,23]. This instrument was equipped with an attenuated total reflectance (ATR) sampling accessory. The ATR ZnSe cell was carefully cleaned with pure isopropanol to eliminate the presence of residues between measurements. Sample spectra were registered using as a background spectrum obtained in the air with a dry empty ATR cell. Samples spectra were collected at a resolution of 4 cm^−1^ and 32 scans per sample vs. background, using OPUS software (BrukerOptik GmbH, Ettlingen, Germany). 

### 2.3. Research Regarding Antioxidant Assays 

As stated in the introduction, a high antioxidant potential is beneficial for an antiangiogenic action required in cancer therapy.

In our case, the antioxidant effects were determined through in vitro tests by using spectrophotometric methods: total antioxidant capacity, ferric reducing power, and radical scavenging ability. For this, a UV-VIS Spectrophotometer UV-1900, (Shimadzu Corporation, Kyoto, Japan), was used. Similar methods were used in one of the previous studies conducted on polymer matrices in the form of films [14]. In the case of nanoparticle samples, after adding the reagents, they were first centrifuged and subsequent determinations were made using the corresponding supernatant.

Thus, the antioxidant capacity was determined based on the phospho-molybdenum method, which includes the preparation of a each nanoparticles stock solution (5 mg/mL in 2% acetic acid), of which 50 μL will be mixed with 2 mL reagent solution. The reagent contains the following substances: sulphuric acid 0.6 M, sodium phosphate 28 mM and ammonium molybdate 4 mM. The absorbance of the sample was measured at 695 nm, using a blank which contains 2% acetic acid (50 μL) and the reagent solution (2 mL); effective concentrations (EC50) were calculated [24,25]. 

Ferric reducing power is a method that involves the use of potassium ferricyanide, trichloroacetic acid and ferric chloride as reagents. In this regard, 1 mL of stock solution (5 mg/mL in 2% acetic acid) was mixed with 1 mL of sodium phosphate buffer (0.2 M, pH = 6.6) and then 1 mL of 1% w/v potassium ferricyanide was added, followed by samples incubation for 20 minutes at 50 °C. To stop the reaction, 1 mL of trichloroacetic acid (10% w/v) was added. The absorbance of each sample was measured at 700 nm, using a blank that contains all reagents and less stock solution. The effective concentrations (EC50) were calculated for this method (also [24]). 

Radical scavenging ability has been evaluated by using a methanol solution of 1,1-diphenyl-2-picrylhydrazyl (DPPH, 0.1 nM). The samples, 50 μL of stock solution (5 mg/mL in 2% acetic acid), were left in the dark for 30 minutes, in contact with 2950 μL DPPH solution and, afterwards, the absorbance at 517 nm was measured using DPPH in methanol as a control solution [25]. The next formula was used for this test:

% inhibition (I%) = (A_c_-A_s_)/A_c_ × 100 (1)

where A_c_ is the absorbance of the control and A_s_ for the sample. 

All the experiments were performed in triplicate.

### 2.4. In Vivo Evaluation of Angiogenic Activity Using a CAM Model

The CAM model was used to assess the angiogenic activity of chitosan derivatives’ nanoparticles. The main functions of CAM are: respiratory organ for the embryo, storage membrane of excretions and also membranes for mobilization of calcium from the shell to start bone mineralization [17]. The CAM assay was performed according to Kue C.S., et al., 2015 and Theng Ng C., et al 2018 [26,27] with some modifications. For this purpose, hen eggs were incubated at 37 °C under 60–70% relative humidity. Furthermore, nine-day-old fertilized chick eggs were selected, and a small window of 1 cm^2^ was made in the shell. Sterile disks of methylcellulose loaded with chitosan derivatives nanoparticles (100 μg/disc) were placed at the junction of two big vessels. The windows were sealed with tape, and the eggs prepared as such, were incubated at 37 °C for 72 h [28]. The embryos were considered dead if they were motionless and had cloudy contents. At the end, CAMs were fixed with 4% paraformaldehyde at 4 °C overnight, and angiogenesis was determined by counting the number of vessels contacted to disks with nanoparticles. The eggs with nanoparticles were compared with those without nanoparticles, in order to observe the new vessel formation.

Histopathological study. In order to evaluate the effect of the presence of nanoparticles on the development of angiogenesis, a histopathological study was performed. The first step was to include CAM membrane fragments taken in paraffin. The obtained paraffin blocks were cut into the microtome at 2–3 μm thick, and furthermore the sections were specifically stained with haematoxylin and eosin H & E [29]. The microscopic examination was performed using a Nikon Eclipse 50i microscope. 

For the microscopic exam, each slide was scanned, following the same exposure and calibration procedure. The sections from the identified region which were scanned received a standard name and were outlined. The morphometric study was realized on representative fields of view (FOV) acquired in 5 distinct regions of the digital images, following the same rule of monitoring the structures distributed central and in the four extremities of each FOV. The blood vessels were counted in each distinct FOV/sample and the results were included in our database.

### 2.5. In Vitro Cytotoxicity Evaluation Using an MTT Assay

In vitro samples cytotoxicity evaluation was performed according to Standard SR-EN ISO 10,993 for cell viability determination, using an MTT assay. The morphology of the cells was examined under the optical microscope after 48 h of samples treatment. 3-(4,5-dimethyl-thiazol-2-yl)-2,5-diphenyltetrazolium bromide (MTT) was purchased from Sigma Aldrich (Ettlingen, Germany).

#### 2.5.1. Cell Cultures

The cell lines used for cytotoxicity evaluation were, on the one hand, NCTC clone L929 cell culture of mouse normal fibroblasts and Hep-2 tumor cell culture of human cervix epithelial cells, on the other hand. Both types were purchased from ECACC (Sigma-Aldrich, Merck Group, Darmstadt, Germany), together with Minimum Essential Medium (MEM), L-glutamine, and antibiotics penicillin, neomycin and streptomycin. Fetal bovine serum (FBS) was provided by Biochrom (Cambridge, UK). 

The cells were cultivated in MEM, supplemented with 10% FBS, 2 mM L-glutamine and a mixture of 100 U/mL penicillin, 100 μg/mL streptomycin, and 500 μg/mL neomycin. In the experiments, the cells were seeding at the density of 4 × 10^4^ cells/mL for normal fibroblasts NCTC, respectively 6 × 10^4^ cells/mL, for cervix epithelial Hep-2 cells, in 24-well culture plates incubated at 37 °C in a humid atmosphere. Then, the samples (sterile disks of methylcellulose loaded with chitosan derivatives nanoparticles (100 μg/disc)) were added and the plates were incubated for another 24 h, respectively 48 h, at 37 °C and 5% CO_2_ in a humid atmosphere. The control of each experiment consisted of an untreated cell culture, while the positive control was a solution of 0.003% H_2_O_2_ in medium. Each experiment was carried out in triplicate.

#### 2.5.2. Cell Morphology Examination at 48 h of Treatment with Samples

After 48 h of treatment, the morphology of the cells was examined using a Zeiss optical microscope AXIO-Observer D1 (Germany) and images of the cell morphology were taken.

*Cell viability* was determined using the MTT colorimetric method. Its principle consists of: tetrazolium salt reacted with mitochondrial dehydrogenases, resulting in formation of insoluble blue-violet formazan crystals and soluble in isopropanol [30]. 

The MTT assay was performed at intervals of 24 and 48 h, after the removing of the samples from the wells, the culture medium was replaced with the tetrazolium salt solution, followed by culture plates incubation for 3 h at 37 °C and 5% CO_2_ in a humid atmosphere. The absorbance of the resulting isopropanol solutions was measured using a Mithras LB 940 Multimode Microplate Reader from Berthold Technologies (Germany), at 570 nm wavelength. At the end, cell viability was determined using the next formula:

(2) $$\%\;cell_{viability}=O.D.\frac{sample}{control}*100\;\%,$$

where O.D is the optical density and the control considers 100 percentage points

### 2.6. Statistical Methods 

The obtained data were processed using the statistical functions of SPSS 18.0 at the 95% significance threshold. In calculating the significant difference between two or more groups, we applied the following:

- one way ANOVA was conducted, for the variables X = blood vessels and Y = study batch, using an alpha of 0.05;
- for multiple comparisons of normal distributed series of values, a post-hoc Bonferroni test was applied after one-way ANOVA in case of significance. One-Way ANOVA was significant (alpha of 0.05) and, in these conditions, Bonferroni correction (post-hoc Bonferroni) was performed to reduce the error rate when testing multiple hypotheses.
- Skewness (−2 < *p* < 2) tests are tests of normality in frequentist statistics, available when using the distribution platform to examine a continuous variable.

In the same descriptive table of the series of values, the homogeneity of these series was demonstrated (Skewness test) and multiple comparisons were made (between several study groups) of the average values—the F test (ANOVA) is used when comparing 2 or more mean values from groups with normal distributions. 

## 3. Results

### 3.1. Chitosan-Derivatives Nanoparticles: DLS Measurements

**Figure 1:**
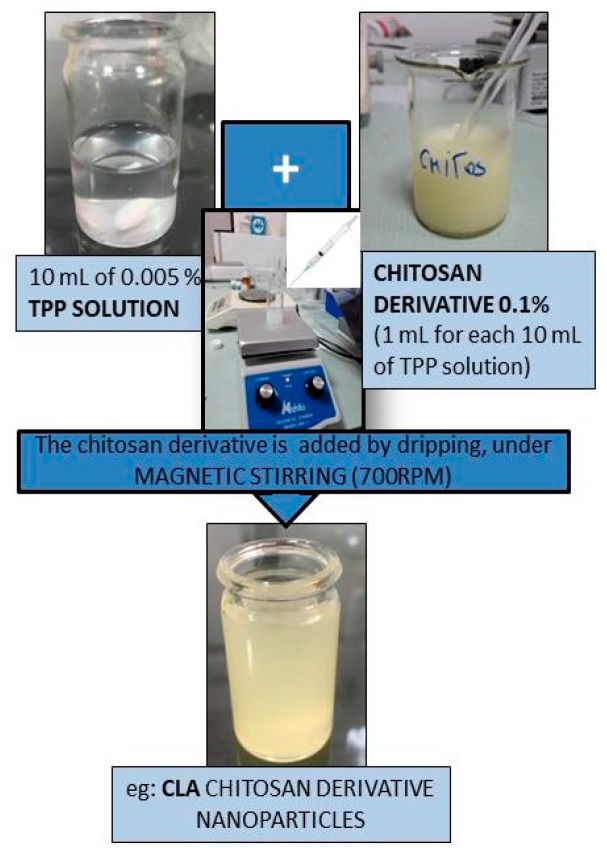
Obtaining scheme of *CLA nanoparticles*.

The obtaining scheme of CLA nanoparticles is presented in Figure 1. The DLS measurements, made for nanoparticles of chitosan derivatives of low molecular weight (CLA, CLB, CLC, CLD), obtained under the conditions of using 10 mL 0.005% TPP solution, are shown in the Table 1. According to the obtained results, the measured sizes for chitosan derivatives nanoparticles are between 351 and 622.2 nm.

Some examples of the DLS measurements of the chitosan derivatives nanoparticles (the size distribution by intensity) are presented in Supplementary Materials.

### 3.2. FT-IR Characterization of Nanoparticles

**Figure 2:**
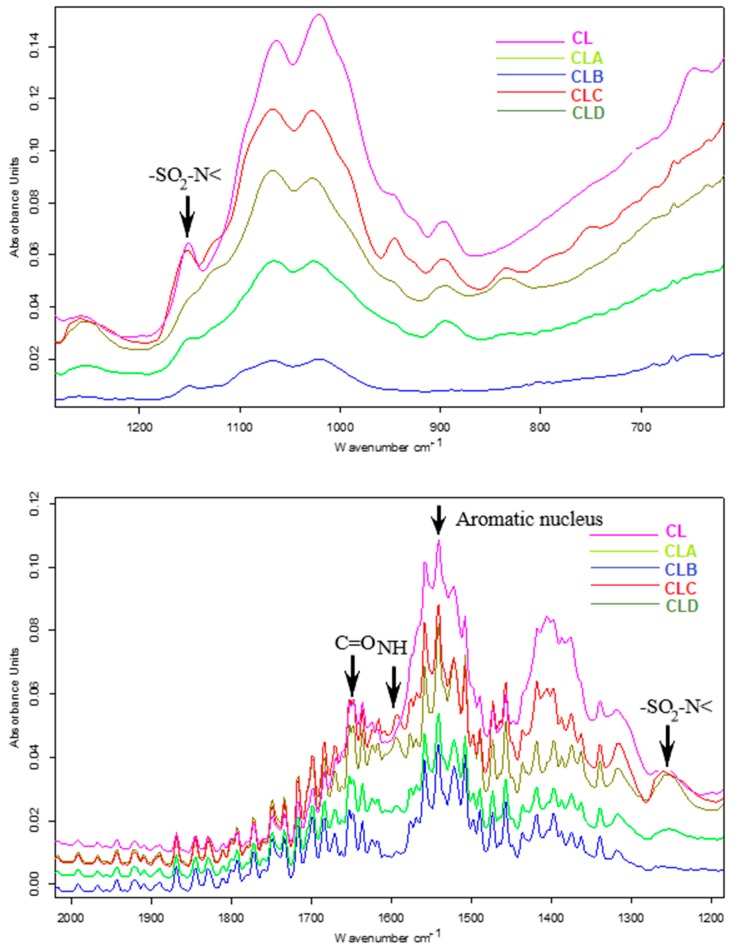

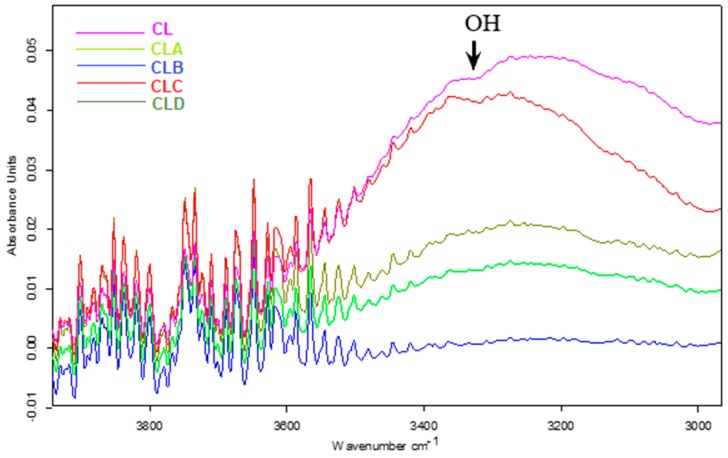
FT-IR characterization spectra of chitosan based nanoparticles and its derivatives (CL, CLA, CLB, CLC, and CLD).

Thus, in the FT-IR spectra of chitosan nanoparticles (Figure 2) as well as that of its functionalized derivatives (CLA-CLD), the characteristic bands of the amide group have been identified, due to vibrations C=O (amide I) in the range 1645–1658 cm^−1^ and NH (amide II) in the range 1594–1598 cm^−1^. At the same time, in the region 3320–3400 cm^−1^, a broad band was identified attributed to the valence vibration of the alcoholic OH groups. The sulfonamide component is present in the spectrum through the absorption bands characteristic of the sulfonamide group, -SO_2_-N< in the 1256–1260 cm^−1^ and 1162–1165 cm^−1^ ranges. The aromatic nucleus is present through multiple bands, of which the most intense are those from regions 1542–1551 cm^−1^. The bands were assigned according to the data from the literature [15,31] and support the idea of including in the nanoparticle matrix, the four derivatives with sulfonamide structure.

### 3.3. Researches Regarding Antioxidant Assays

The new derivatives of chitosan with sulfonamidic structure were evaluated from the point of view of antioxidant potential.

#### 3.3.1. Determination of Total Antioxidant Activity (TAA)

The total antioxidant activity was determined using phosphomolybdenum blue complex with a maximum absorption at 695 nm.

**Figure 3:**
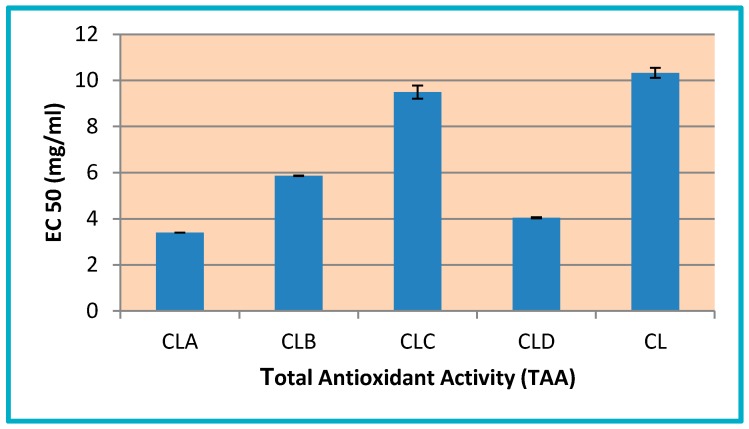
Total antioxidant activity (EC50 mg/mL) of chitosan nanoparticles and its derivatives (CL, CLA-CLD).

EC50 values of chitosan low molecular weight derivatives are comparable (between 3.4 and 9.49 mg/mL) with the one of pure chitosan (10.32 mg/mL). In this context, the most active compounds are: CLA with EC50 = 3.40 mg/mL (three times more active than pure chitosan) and CLD with EC50 = 4.04 mg/mL (2.5 times more active than chitosan)—Figure 3.

#### 3.3.2. Ferric Reducing Power

The measurement of reducing power is particularly important for the antioxidant activity of the compounds. The presence of a reducing agent in the obtained nanoparticles results in reduction of the ferric/ferricyanide complex to its ferrous (Fe^2+^) form.

**Figure 4:**
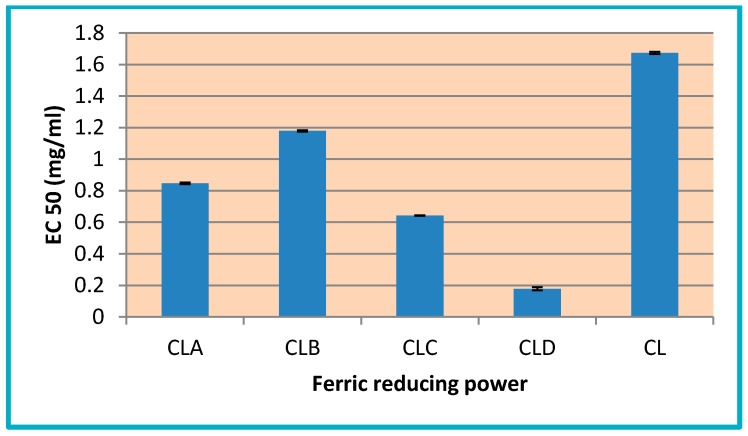
Ferric reducing power (EC50 mg/mL) of chitosan nanoparticles and its derivatives (CL, CLA-CLD).

The values obtained from this assay (EC50 values between 0.1783 and 1.179 mg/mL) demonstrate that chitosan derivatives nanoparticles (CLA-CLD) are more active than pure chitosan CL (with EC50 = 1.67 mg/mL) (Figure 4), the amount of Fe^2+^ being monitored at 695 nm, by measuring the intensity of Perl’s Prussian blue color complex.

#### 3.3.3. Radical Scavenging Ability

DPPH is a radical, which demonstrates a strong absorption band at 517 nm, and it passes into colorless or pale yellow, if it is neutralized by antioxidant substances. Thus, this radical is commonly used to evaluate the radical scavenging capacity of antioxidants.

**Figure 5:**
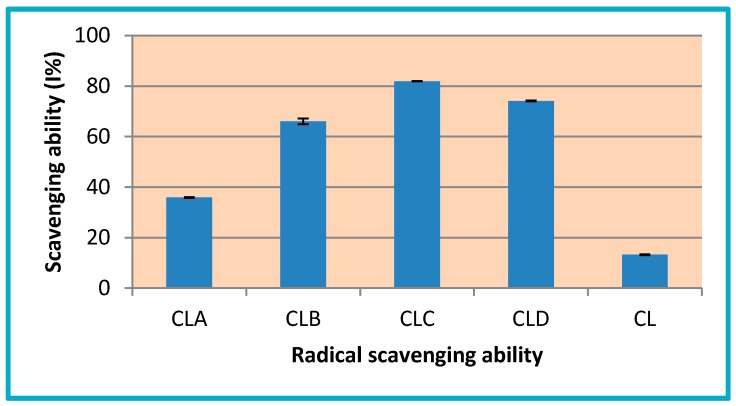
DPPH radical scavenging ability (I%) of chitosan nanoparticles and its derivatives (CL, CLA-CLD).

Chitosan derivatives (CLA-CLD) have a good scavenging ability with I = 35.86–81.94%, while, for pure chitosan (CL), this value is 13.21%. The most active compounds are CLC (I% = 81.94%) and CLD (I% = 74.13%), these being 6.2 and 5.6 times more active than pure chitosan (Figure 5).

The results of antioxidant potential evaluation, through in vitro tests (total antioxidant capacity, reducing power and inhibiting capacity of free radicals DPPH), confirmed the fact that nanoencapsulation of all chitosan derivatives have positively influenced their antioxidant potential, these being more active than pure chitosan.

### 3.4. In Vivo Evaluation of Angiogenic Activity Using a CAM Model

**Figure 6:**
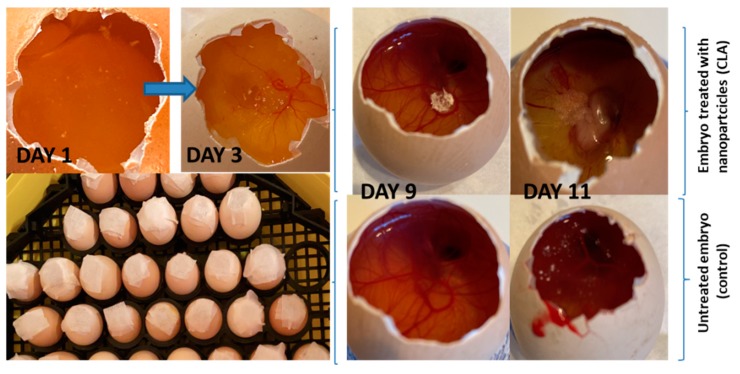
CAM Angiogenesis Model: macroscopic images (after 1, 3, 9, and 11 days of incubation in the presence (CLA) and in the absence of nanoparticles (CL—control)).

Figure 6 shows macroscopic images obtained during the 11 days of incubation. The images show different stages of the embryos, in the absence and in the presence of the tested samples.

The total number of blood vessels tested/field has the following particularities (Table 2):

- variations from 0 to 8 vessels/field;
- mean 3.88 ± 2.28 vessels/field;
- the median value (4 vessels/field) close to the average value and the Skewness test result <2 suggests that the series of values of the number of vessels was homogeneous, so statistical significance tests can be applied.

**Figure 7:**
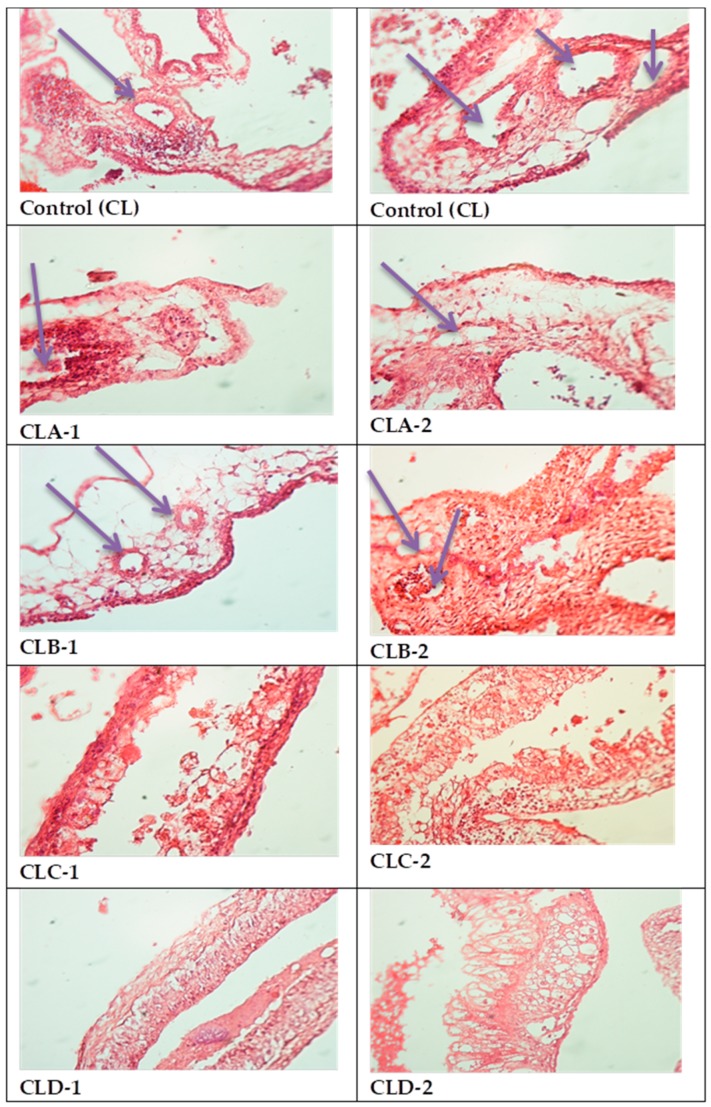
CAM Angiogenesis Model (microscopic images)-**Control (CL):** Abundant vascularization dispersed in mesenchyme; **CLA-1, CLA-2:** Vascular network present, but large mesenchymal areas do not present vasculature; **CLB-1, CLB-2:** Very small variability of vascular supply; **CLC-1.CLC-2**: Significant decrease of blood vessels presence; **CLD-1, CLD-2:** Significant decrease of vascular support.

**Figure 8:**
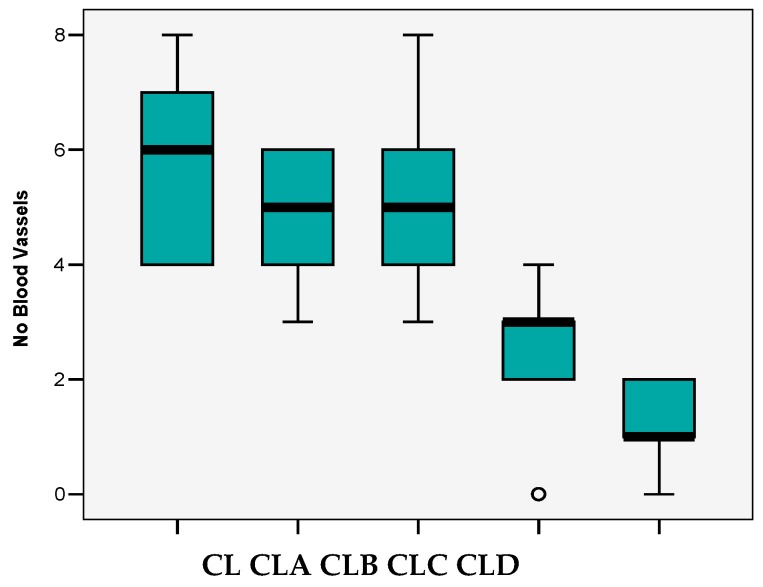
The average number of blood vessels/field.

All four types of nanoparticles resulted in reduced angiogenesis (Figure 7), but the maximum effect was observed in CLC and CLD cases, with significant decrease of vascular support (Table 2, Figure 8).

Thus, in the case of CL control, the median value of the blood vessels registered in the field was 6, which decreased significantly in the case of CLC (*p* < 0.05) and CLD (*p* < 0.001) nanoparticles. In these cases, the values achieved were three and one vessels/field. In addition, in the case of CLA and CLB samples, there were slight decreases of these values (5 vessels/field) (*p* > 0.05) (“*p*” is corresponding to the correlation between the average level of the vessels and the groups referred to the control (CL)).

### 3.5. In Vitro Biocompatibility Evaluation Using MTT Cell Viability Assay

MTT assay results on normal mouse fibroblasts NCTC at 24 and 48 h test intervals indicated good biocompatibility of all tested samples (100 μg/disc), at 24 h cell viability values for samples CLA, CLB, CLC, and CLD were between 98.07 and 105.79%, higher compared to the viability obtained in the reference sample CL (95.72%), in a similar way at 48 h of experiment the samples CLA, CLB, CLC and CLD registered viabilities values of 99.28–107.52%, higher than the cell viability 89.29% of the reference sample. The testing in NCTC normal cells at 48 h indicated a good biocompatibility of all tested samples, with values close or higher than the control, with highest viability values for samples CLC and CLD (106.18%. and 107.52%)—Table 3.

**Figure 9:**
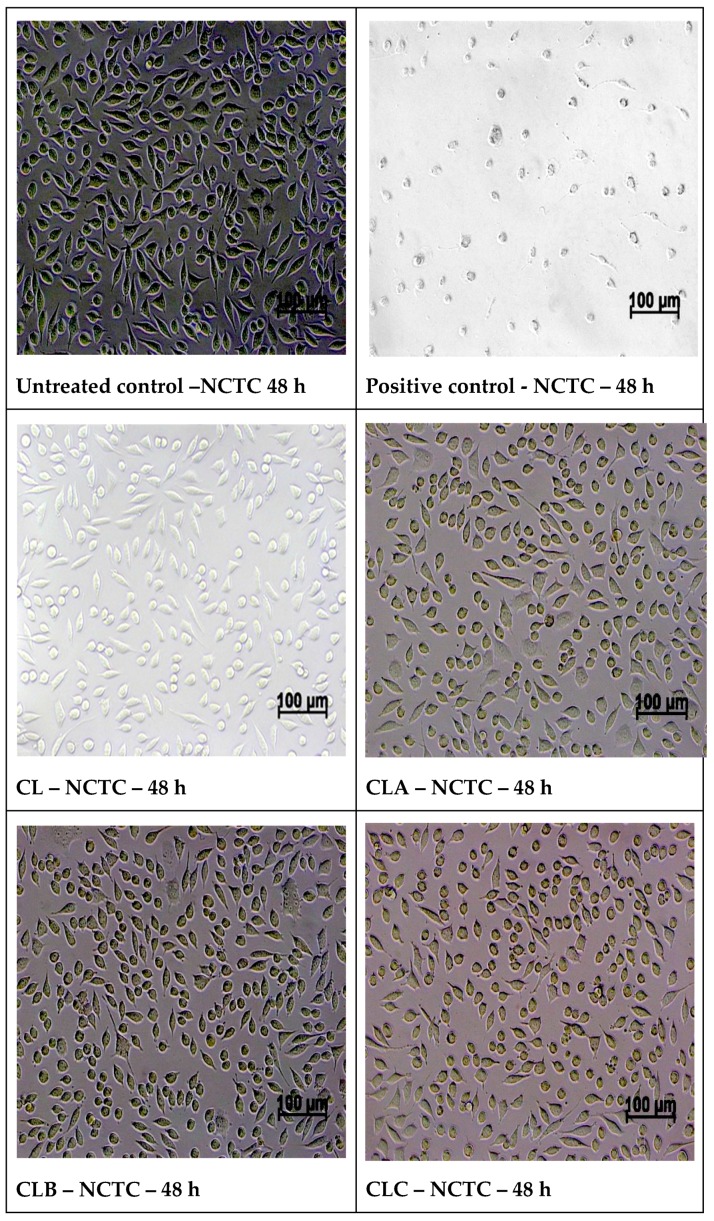

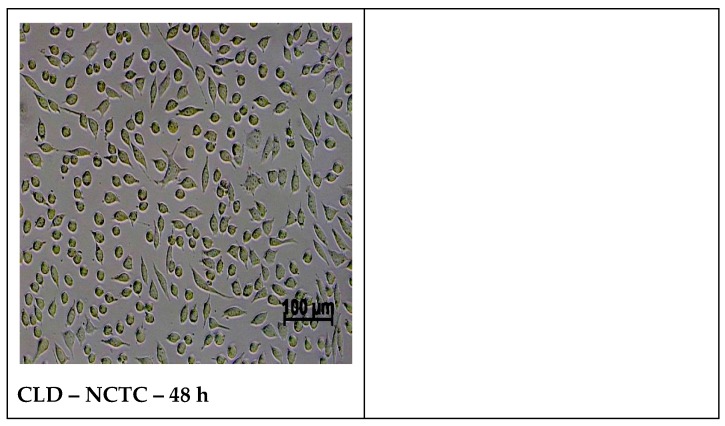
Optical microscopy images of normal NCTC fibroblast cells, after 48 h of direct contact with nanoparticles samples.

Optical microscopy images of normal NCTC fibroblast cells, after 48 h of direct contact with nanoparticles samples, present a morphology similar to control NCTC cell culture, with cells of fusiform or round conformation, with fine intracytoplasmic granules, and having a cell density similar or slightly variable to the control (Figure 9).

### 3.6. In Vitro MTT Assay on Human Epithelial Cervical Tumor Hep-2 Cells

From MTT assay on human epithelial cervical tumor cells Hep-2 at 24 h, all the viability results of the samples were higher than 85%, indicating lack antitumor activity. At 48 h of testing, the reference sample CL with 87.85% viability had no antitumor effect, while samples CLB and CLC with viability of 79.69% and 69.91% present a weak antitumor activity. The samples CLA and CLD recorded 66.16, respectively, 66.89% viability, showing the most evident antitumor activity on Hep-2 tumor cells (Table 3), for the tested concentration (100 μg/disc).

**Figure 10:**
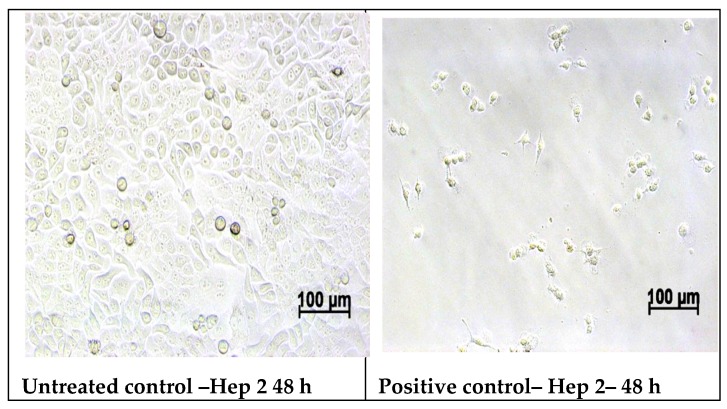

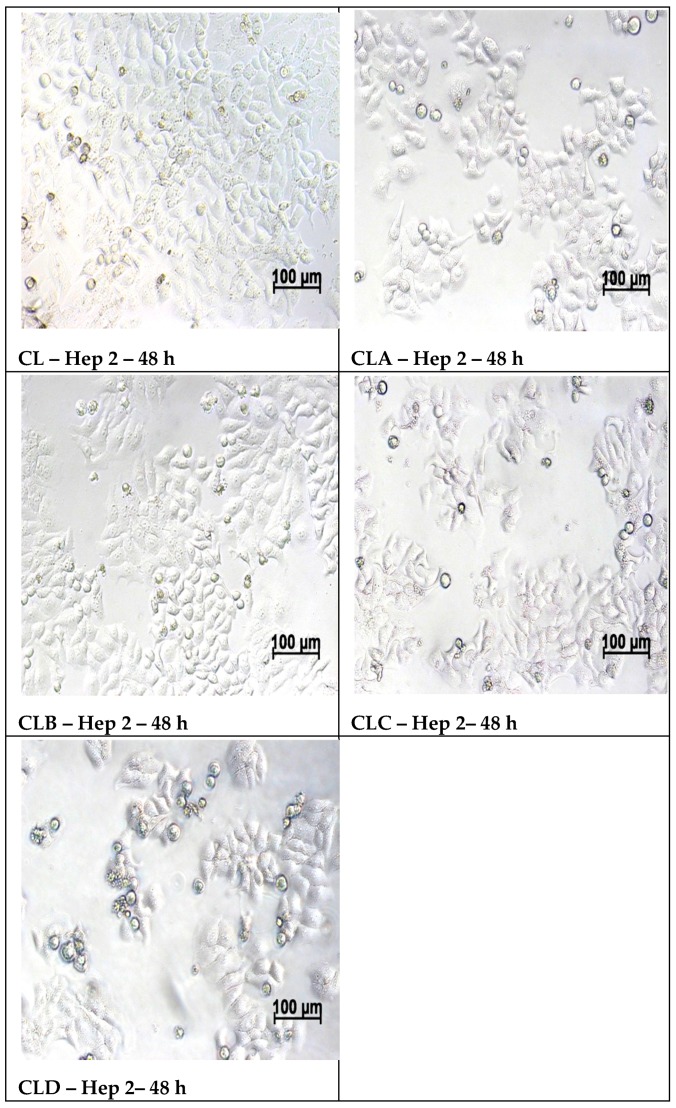
Optical microscopy images of the Hep-2 tumor cells after 48 h of treatment with nanoparticles’ samples.

The optical microscopy images of the Hep-2 tumor cells after 48 h of treatment with nanoparticles samples, showed that the cells treated with the CL reference sample have similar aspect with cell morphology of the control culture, with cells of polygonal shape and regular size, indicating the lack of antitumor effect of the sample on Hep-2 culture. In contrast, the images of the cells treated with samples CLA, CLB, CLC and CLD, shows numerous round and/or lysed cells in the culture, or cells detached from the surface of the well as result of an extended cell lysis, indicating the antitumor activity of the respective samples (Figure 10).

Optical microscopy images of the morphology of normal NCTC cells and respectively Hep-2 tumor cells treated with tested samples confirm the cell viability results obtained by the MTT assay.

## 4. Discussion

Until recently, scientists believed that, due to the rapid development of tumors, they become too numerous to be supplied with blood and, as a result, they die quickly. In fact, cancerous tumors stimulate the development of an entire bundle of new blood vessels that supply the nutrients they need. This process is called angiogenesis, and animal and cell culture studies have shown that, if this process is interrupted, the tumor will "starve." In vitro and in vivo investigations show that, in the absence of blood vessels, tumors can grow until passive diffusion can no longer ensure the supply of nutrients and the elimination of degradation products in the adjacent environment. Tumor angiogenesis results from the interaction of three types of cell populations: tumor cells, stromal cells (macrophages, mast cells), and endothelial cells [32]. By the end of the 1990s, most of the drugs used in cancer therapy were acting through incisive mechanisms, particularly targeting the inhibition of DNA replication of tumor cells undergoing excessive and uncontrolled mitosis, leading to apoptosis. Chemotherapy of this kind has the disadvantage of non-selectivity, also entailing many healthy cells, the more exposed being those with accelerated turnover, but, fortunately, the effect on cancer cells is greater [33].

In general, in normal conditions, reactive oxygen species, such as superoxide (^·^O_2_^−^), peroxides (H_2_O_2_), hydroxyl radicals (·OH), are maintained in basal levels, while it is well known that tumor cells produce higher levels of H_2_O_2_. Moreover, studies incriminate peroxides by stimulating pathological angiogenesis. In this respect, different researchers have demonstrated in their studies that antioxidants such as catechins [34], resveratrol [35], polyphenols [36], flavonoids [37], nutritional components, and also synthetic compounds [38] are able to inhibit angiogenesis.

In addition, our research is focused on the antioxidant action of chitosan-sulfonamide derivatives (CLA-CLD) on the one hand, but also on the potential of nanostructures to inhibit angiogenesis through the large contact surface that they provide. According to our findings, free low molecular weight chitosan has a much lower antioxidant activity comparing to its derivatives, this being demonstrated both by the existence of higher effective concentrations and by lower percentages of the ability to inhibit free radicals.

In conventional drug discovery, preclinical animal models which are used to investigate the pharmacological properties of new bioactive substances have the disadvantage of being expensive and time-consuming [39]. However, starting with 1959, in the fundamental research, the principle of the “Three Rs” was taken into account, to reduce the suffering of animals involved in pharmacological studies. In this context, the first R refers to the “replacement” of laboratory animals with computer models or in vitro cultures, but also with smaller animals that have a lower potential for pain perception. Thus, for our study, we have chosen the CAM model, which is an intermediate model between cell-based and animal-based assays, the advantage of such model being that it also gives tissue responses similar to those in mammalian models [26]. Furthermore, the Institutional Animal Care and Use Committee (IACUC) [40], an Association of New England Medical Center and Tufts, as well as the National Institutes of Health USA, mandated that *<a chick embryo that has not reached the 14th day of its gestation period would not experience pain and can therefore be used for experimentation without any ethical restrictions or prior protocol approval>,* which is an advantage. This test also ensures compliance with the second R, which refers to “refinement” to minimize animal suffering, while in terms of the third R, this test would be helpful in “reducing” the number of animals involved [26].

The CAM model is a quick and low-cost test that allows analysis of a large number of pharmacological samples in a short period of time. Kue et al. [26] have applied this method to study the acute toxicity of approved anti-cancer drugs (like cisplatin, vincristine, cyclophosphamide, etc.). The results of their study show that there is a significant correlation between LD50 values generated using the CAM versus mice and therefore the test could be used in preliminary studies to reduce testing time and the number of animals. Ng et al. [27] have used in their study Suramin as a positive control to inhibit vessel sprouting in a CAM model. They have used this standard to demonstrate that *Clinacanthus nutans* water extract prevents new blood vessel formation in the CAM model. Suramin is known for its antiangiogenic activities, being involved its antioxidant properties, which were found to be higher than in the case of l-ascorbic acid. In this regard, studies have shown that Suramin treatment increases the activity of antioxidant enzymes in the tissues (such as SOD, CAT, GST, GPx and GR) [41].

In our case, the chorioallantoic membrane model underlined the antiangiogenic effect of antioxidant nanoparticles, the most powerful effect being established for CLC and CLD nanoparticles (with three and one vessels/field, respectively) (which have also demonstrated their strongest antioxidant action), these being the corresponding nanoparticles of chitosan-sulfadimethoxine and chitosan-sulfisoxazole derivatives. Our finding, regarding the fact that the isoxazolic nucleus (for the CLD derivative) and the dimethoxylated aromatic nucleus (for the CLC derivative) generate the most active nanoparticles, is also sustained in the context in which the studies in this field have reported a naturally occurring diarylisoxazole derivative, as a new chemical tool with efficacy against breast cancer cells 22. In addition, in their study, Çalışkan et al. [42] have synthesized new isoxazole-piperazine hybrids and analyzed their cytotoxic activities on human cancer cell lines. In this study, the isoxazole derivatives showed moderate to significant cytotoxicity on the used cell lines. As for our dimethoxylated derivative (CLC- chitosan-sulfadimethoxine), the existence of methylated OH groups leads to more potent nanoparticles in the ability to kill cancer cells by apoptosis, as Chen et al. have proven in their study for dimethoxy curcumin [43].

In the case of the other derivatives, for which the aromatic nucleus is substituted with a single methoxy group (CLA-Chitosan-sulfamethoxydiazine), or even unsubstituted (CLB- Chitosan-sulfadiazine), the antioxidant action is weaker, recorded by higher EC 50 and lower percentages of DPPH radical scavenging ability (I%), compared to other derivatives, which also leads to a lower ability to inhibit angiogenesis, proven by the CAM test. Thus, in the case of these derivatives, if, for the antioxidant action EC values of up to 1179 mg/mL and DPPH radical scavenging ability of up to 35.86% being recorded for the inhibition effect of angiogenesis, five vessels/field were determined.

In addition, Hep-2 tumor cells indicated that all chitosan derivatives induced antitumoral effect. At a concentration of 100 μg/disc, all four types of nanoparticles were biocompatible in NCTC cells (with a cell viability of over 99%), while, in Hep-2 tumor cells cultivated in medium, these nanoparticles were anti-proliferative (with a viability of up to 66%) after 48 h of treatment.

Regarding the high in vitro biocompatibility of new chitosan derivatives, processed as nanoparticles, due to chitosan, extracted from the exoskeleton of crustaceans, whose non-toxicity, biocompatibility, and bioactivity is well known in the literature, these nanostructures show good biocompatibility, slightly increased compared to those generated by pure chitosan, from 89.29% to over 100%. In one of our previous studies [15], in which such chitosan-sulfonamide derivatives were processed under the form of sponges for the treatment of burns, we demonstrated a relatively low biocompatibility of these compounds, using the same mouse fibro-blasts (L929), with cell viability at 48 h between 56.86% and 74.34%. It appears that our nanostructures had a better cell biocompatibility, close to that of untreated control (100%).

In terms of sulfonamides toxicity, Zessel et al. demonstrated in one of his studies [44], using murine fibroblasts and keratinocytes, that this class in general, and the sulfonamides used by us, in particular, have no impact on cell toxicity.

Hep-2 tumor cells treated with tested samples show a beneficial effect for the modified chitosan with the four sulfonamides and for nano-encapsulation of chitosan derivatives, by recording lower cell viability values compared to the parent chitosan. The MTT assay results for tested nanoparticles in Hep-2 cells exerted a strong cytotoxity at 100 μg/disc (between 66.16% and 79.70% viability). In addition, it was observed that pure chitosan had no significant antitumor activity at this concentration (only 87.85% viability).

## 5. Conclusions

Currently, the medical relevance of nanomaterials for the prevention and treatment of human cancer remains a continuing challenge. However, the use of appropriate nanostructures, capable of overcoming biological barriers, could be an important strategy for future antitumor therapy. In our study, four types of chitosan derivatives nanostructures were biocompatible for normal NCTC cells (more than 99% viability) and cytotoxic for Hep-2 tumor cells (up to 66% viability), as confirmed by in vitro experiments. In addition, their antioxidant action, demonstrated by three in vitro tests, had a beneficial impact on their ability to inhibit angiogenesis, demonstrated by the CAM test, reducing vascularization from six vessels per field, in the case of pure chitosan (CL), to one vessel per field, in the case of the CLD derivative. In conclusion, our results demonstrate that chitosan derivatives’ nanostructures strongly enhance the therapeutic effect of free chitosan, as far as their antitumor potential is concerned.

## Figures and Tables

**Table 1:** Size of chitosan-sulfonamide nanoparticles.

Compound	CL (chitosan)	CLA	CLB	CLC	CLD
Dimension (nm)	390.2	622.2	571	351	435

**Table 2:** Statistical indicators of the number of blood vessels/field compared to study groups.

		All Groups	CL	CLA	CLB	CLC	CLD
N		25	5	5	5	5	5
Mean		3.88	5.80	4.80	5.20	2.40	1.20
p post-hoc Bonferroni				ns)	ns) ns)	b) ns) ns)	a) b) b) ns)
Median		4.00	6.00	5.00	5.00	3.00	1.00
Std.Deviation		2.28	1.79	1.30	1.92	1.52	0.84
Variance		5.19	3.20	1.70	3.70	2.30	0.70
Skewness Test		0.092	0.052	−0.541	0.590	−1.118	−0.512
Std.Err.Skewness		0.464	0.913	0.913	0.913	0.913	0.913

a) *p* < 0.001 b) *p* < 0.05 ns) *p* > 0.05.

**Table 3:** Cell viability values resulted from MTT in vitro assay at 24 and 48 h of treatment with nanoparticles.

Cell Culture	Time	Untreated Control	Positive Control	CL	CLA	CLB	CLC	CLD
**NCTC**	**24 h**	100%	11.45%	95.72%	105.79%	100.00%	98.07%	104.83%
	**48 h**	100%	7.31%	89.29%	99.28%	101.96%	107.52%	107.52%
**Hep-2**	**24 h**	100%	13.86%	113.69%	85.00%	111.96%	99.35%	98.15%
	**48 h**	100%	6.95%	87.85%	66.16%	79.70%	69.91%	66.89%

## Supplementary Materials

The following are available online at https://www.mdpi.com/2079-4991/10/4/698/s1, Figure S1: DLS measurements: The size distribution by intensity of chitosan derivatives nanoparticles (a. CLA; b. CLB; c. CLD); Figure S2: Histogram of the number of blood vessels/field.
