# Supplementary material for: Designing of Chitosan Derivatives Nanoparticles with Antiangiogenic Effect for Cancer Therapy
Source: Nanomaterials (Basel). 2020 Apr 7;10(4):698. doi: 10.3390/nano10040698 (PMC7221956; doi:10.3390/nano10040698)
Supplement: Supplementary file 1 [file nanomaterials-10-00698-s001.pdf]

nanomaterials

Supplementary material

Antiangiogenic effect with antitumoral potential of antioxidant chitosan nanoparticles, on Chick Chorioallantoic Membrane

Oana-Maria Dragostin[1*#], Rodica Tatia[2#], Sangram Keshari Samal[3#], Anca Oancea[2], Alexandra Simona Zamfir[4], Ionuț Dragostin[4], Elena-Lăcrămioara Lisă[1], Constantin Apetrei[5*], Carmen Lăcrămioara Zamfir[4]

[1] Research Centre in the Medical-Pharmaceutical Field, Faculty of Medicine and Pharmacy, "Dunarea de Jos" University of Galati, 800008, Galati, Romania.; oana.dragostin@ugal.ro ; elenalysa@yahoo.com

[2] Romanian National Institute of Research and Development for Biological Sciences, 296 Splaiul Independentei, 060031 Bucharest, Romania; rodica.tatia@gmail.com

[3] Laboratory of Biomaterials and Regenerative Medicine for Advanced Therapies, Indian Council of Medical Research-Regional Medical Research Centre, Bhubaneswar 751 023, Odisha, India; sksamalrec@gmail.com

[4] Department of Morpho-Functional Sciences I, Faculty of Medicine, University of Medicine and Pharmacy "Gr.T.Popa" Iasi, Romania; simona.zamfir14@gmail.com; ionut.dragostin@yahoo.com; zamfircia@yahoo.com

[5] Department of Chemistry, Physics and Environment, The European Centre of Excellence for the Environment, Faculty of Sciences and Environment, "Dunarea de Jos" University of Galati, 800008 Galati, Romania; apetreic@ugal.ro

* Correspondence: oana.dragostin@yahoo.com (O.M.D) ; apetreic@ugal.ro (C.A)

[#] These authors had equal contributions

Received: 15 March 2020; Accepted: 3 April 2020; Published: 7 April 2020

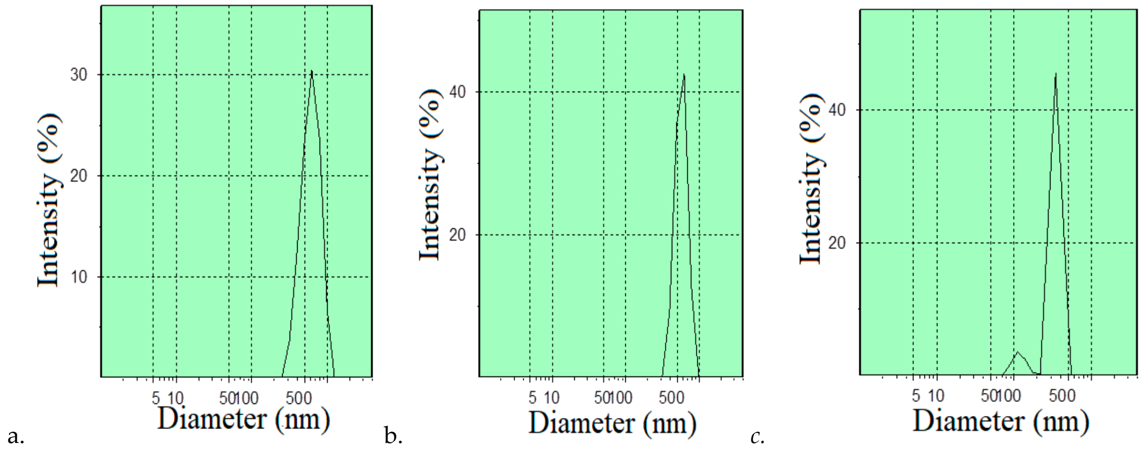

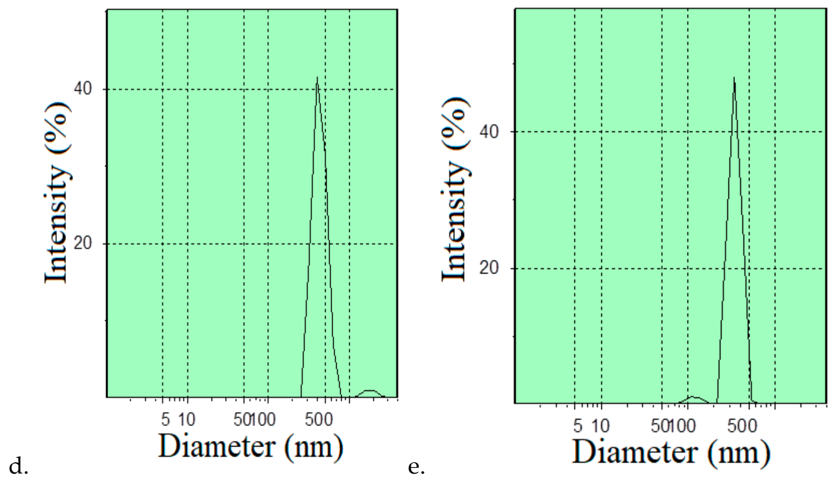

Fig. S1. DLS measurements: The size distribution by intensity of chitosan and chitosan derivatives nanoparticles (a. CLA; b. CLB; c. CLC; d. CLD; e. CL)

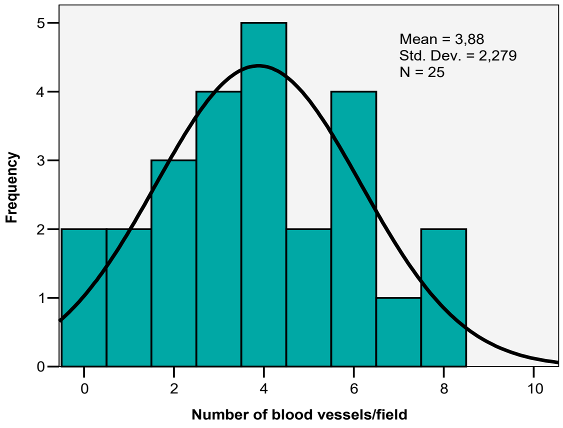

Fig. S2. Histogram of the number of blood vessels / field

© 2020 by the authors. Submitted for possible open access publication under the terms and conditions of the Creative Commons Attribution (CC BY) license (http://creativecommons.org/licenses/by/4.0/).
